# Comparison between minimally invasive spine stabilization with and without posterior decompression for the management of spinal metastases: a retrospective cohort study

**DOI:** 10.1186/s13018-018-0777-2

**Published:** 2018-04-16

**Authors:** Hiroshi Uei, Yasuaki Tokuhashi, Masafumi Maseda, Masahiro Nakahashi, Hirokatsu Sawada, Enshi Nakayama, Hirotoki Soma

**Affiliations:** Department of Orthopaedic Surgery, Nihon University School of Medicine, 30-1 Oyaguchi Kami-cho, Itabashi-ku, Tokyo, 173-8610 Japan

**Keywords:** Spinal metastases, Palliative surgery, Minimally invasive spine stabilization, Posterior decompression, Tokuhashi score

## Abstract

**Background:**

Posterior decompression and stabilization plays significant roles in palliative surgery for metastatic spinal tumor. However, the indication for addition of posterior decompression have not been examined. The purpose of this study was to investigate a retrospective cohort of outcomes of metastatic spinal tumor treated with minimally invasive spine stabilization (MISt) with or without posterior decompression.

**Methods:**

The subjects were 40 patients who underwent MISt using percutaneous pedicle screws for metastatic spinal tumor, including 20 patients treated with stabilization alone (group A) and 20 patients with added posterior decompression (group B). We analyzed baseline characteristics, postoperative survival time, and perioperative factors such as neurological outcomes, Barthel Index, VAS, and rate of discharge to home.

**Results:**

The mean ages were 70 and 66 years old (*P* = 0.06), the mean revised Tokuhashi scores were 7.2 and 5.8 (*P* = 0.1), the mean spinal instability neoplastic scores (SINS) were 10.5 and 9.0 (*P* = 0.04), and the mean Barthel Index for ADL were 65.5 and 41.0 (*P* = 0.06) in groups A and B, respectively. The median postoperative survival time did not differ significantly between groups A and B (12.0 vs. 6.0 months, *P* = 0.09). Patients in group A had a significantly shorter operation time (166 vs. 232 min, *P* = 0.004) and lower intraoperative blood loss (120 vs. 478 mL, *P* < 0.001). Postoperative paralysis (*P* = 0.1), paralysis improvement rate (*P* = 0.09), postoperative Barthel Index (*P* = 0.06), and postoperative VAS (*P* = 0.6) did not differ significantly between the groups. The modified Frankel classification improved from D1 or D2 before surgery to D3 or E after surgery in 4 of 10 cases (40%) in group A and 8 of 8 patients (100%) in group B (*P* = 0.01). Significantly more patients were discharged to home in group A (*P* = 0.02), whereas significantly more patients died in the hospital in group B (*P* = 0.02).

**Conclusions:**

Patients treated without decompression had a shorter operation time, less blood loss, a higher rate of discharge to home, and lower in-hospital mortality, indicating a procedure with lower invasiveness. MISt without decompression is advantageous for patients with D3 or milder paralysis, but decompression is necessary for patients with D2 or severer paralysis.

## Background

Metastatic spinal tumor can destroy the spine, collapse the spine support, and invade and compress the spinal cord and cauda equina, causing pain, paralysis, and impairment of activities of daily living (ADL). Moreover, most cases of metastatic spinal tumor are systemic diseases with limited treatment [1–6]. Therefore, symptomatic treatment is mainly performed, and it is important to select a treatment method that maximizes pain relief and improves paralysis and ADL under limited conditions as early as possible [1–4, 6–9]. Palliative posterior stabilization is selected for most cases, but massive bleeding may occur during preparation of the surgical field, application of decompression, and intratumor resection; and surgical stress cannot be neglected for patients with limited prognosis. Minimization of surgical stress has been attempted using balloon kyphoplasty [10] and minimally invasive spine stabilization (MISt) with percutaneous pedicle screws (PPS) [9, 11–19]. Minimization of the invasiveness of palliative surgery for metastatic spinal tumor is more important than that of surgery for spinal degenerative disease.

Outcomes of MISt have been reported in palliative surgery using PPS for metastatic spinal tumor [9, 13, 14, 17, 19], and other studies have compared conventional posterior decompression and stabilization [11, 12, 15, 16, 18]. Many studies have focused on surgical stress or short-term paralysis improvement, but only a few have evaluated postoperative survival and ADL [12, 18]. Moreover, differences in outcomes between MISt with and without addition of decompression have not been examined. Therefore, in this study, we investigated survival time, neurological recovery, and improvement of ADL in patients with metastatic spinal tumor treated with palliative MISt with and without posterior decompression.

## Methods

### Patient population

This study was a retrospective review of a prospectively collected data from 71 patients who underwent palliative surgery for metastatic spinal tumor from January 2012 to December 2016 at our hospital. The inclusion criteria for MISt with PPS for metastatic spinal tumor were (1) intractable pain due to spinal instability or threat of instability defined by SINS [20], (2) spinal paralysis such as any change in the motor examination, and (3) radiation-resistant cancer such as kidney cancer or thyroid cancer. The exclusion criteria were (1) the case indicated for total en bloc spondylectomy; (2) lesions in the occipital over the cervical region; (3) difficulty confirming the pedicle of the vertebral arch under fluoroscopy or difficulty in PPS insertion; (4) treatable with balloon kyphoplasty; (5) treatable with posterior decompression alone; and (6) life expectancy < 6 months and responsive to narcotic analgesics or markedly responsive to radiotherapy, poor general condition (Karnofsky performance status 3 or poorer), or reduced will to live. When impossible to treat with MISt, patients were treated with conventional posterior decompression and fixation surgery. Finally, 40 patients were enrolled in this study. The study protocol was approved by the Nihon University Hospitals’ Joint Institutional Review Board. All participants provided written informed consent. In our cohorts, patients were divided into two groups: those in whom stabilization with posterior decompression was applied in 20 patients (group B) and those with posterior stabilization alone in 20 patients (group A).

### Outcome evaluation

The evaluation items were (1) baseline characteristics: age, sex, metastatic tumor diagnosis, main level of tumors, preoperative modified Frankel category [21], revised Tokuhashi score [3], Spinal Instability Neoplastic Score (SINS) [20], epidural spinal cord compression scale (ESCCS) [22], preoperative visual analogue scale (VAS) for pain, and preoperative Barthel Index [23]; (2) postoperative survival time; (3) perioperative factors: operation time, intraoperative blood loss, blood transfusion, number of instrumented level, and incidence of perioperative complications; (4) postoperative factors: grade of postoperative paralysis, paralysis improvement by one or more grades on the modified Frankel classification or maintenance of grade E, Barthel Index (postoperative maximum score), VAS at 2 weeks after surgery, postoperative course (discharge to home, transfer to a hospice, in-hospital death), the use of adjuvant therapy (chemotherapy, radiotherapy, and introduction of bone modifying agent (BMA) therapy), reaggravation of paralysis due to tumor enlargement at the surgically treated level, and reoperation. Differences in outcomes between patients treated with and without radiotherapy were investigated in each group. With respect to postoperative adjuvant therapy, we tried to perform radiotherapy after surgery excluding radiation-resistant cancer, as long as it is not inconvenient for treatment of the primary cancer. The criterion for discharge to home was Barthel Index ≥ 70 or availability of sufficient care by family members for cases with Barthel Index < 70. Statistical analysis was performed using SPSS v. 19.0 (SPSS Inc., Chicago, IL, USA). The Kaplan-Meier method was used to estimate postoperative survival, and survival curves were compared using log-rank test. Paired *t* test and Mann-Whitney test were used for continuous variables, and *χ*^2^ test was applied for categorical data. In all cases, significance level was set at *P* < 0.05. The calculation of post-hoc power analysis for baseline characteristics was performed using G*Power software (version 3.1.9.2) [24]. The alpha error probability was set at 0.05.

## Results

The patients included 16 males (80%) and 4 females (20%) (*P* = 0.6), and the mean ages at the time of surgery were 70.0 and 66.3 years in groups A and B, respectively (*P* = 0.06) (Table 1). The primary lesion was the liver in 5 (25%) and 5 (25%), lung in 3 (15%) and 4 (20%), prostate in 1 (5%) and 2 (10%), thyroid in 2 (10%) and 1 (5%), kidney in 2 (10%) and 1 (5%), breast in 1 (5%) and 1 (5%), and others in 6 (30%) and 4 (20%) in groups A and B, respectively. The primary lesion was gastric cancer in 2 (10%) only in group B. The level of the primary lesion was the thoracic and lumbar spine in 8 (40%) and 12 (60%) patients in group A, and 15 (75%) and 5 (25%) patients in group B, with a significant difference between the groups (*P* = 0.02). The modified Frankel classification indicated that paralysis before surgery was more severe in group B (*P* = 0.02). Preoperatively, patients in group A had a higher revised Tokuhashi score (7.2 vs. 5.8, *P* = 0.1), a higher SINS (10.5 vs. 9.0, *P* = 0.04), and a higher Barthel Index for ADL (65.5 vs. 41.0, *P* = 0.06). The preoperative ESCCS (*P* = 0.1) and VAS for pain did not differ between the two groups (*P* = 0.6).

**Table 1:** Baseline characteristics

Characteristic	Group A	Group B	*P* value
	(*n* = 20)	(*n* = 20)	
Age at surgery, mean (SD), years	70.0 (15.0)	66.3 (9.5)	0.06
Sex, *n* (%)			0.6
Male	16 (80)	16 (80)	
Female	4 (20)	4 (20)	
Metastatic tumor diagnosis, *n* (%)			
Liver	5 (25)	5 (25)	
Lung	3 (15)	4 (20)	
Prostate	1 (5)	2 (10)	
Thyroid	2 (10)	1 (5)	
Kidney	2 (10)	1 (5)	
Breast	1 (5)	1 (5)	
Gastric	0	2 (10)	
Others	6 (30)	4 (20)	
Main level of tumors, *n* (%)			0.02
Thoracic	8 (40)	15 (75)	
Lumbar	12 (60)	5 (25)	
Preoperative modified Frankel category, *n* (%)			0.02
A	0	1 (5)	
B	0	2 (10)	
C	2 (10)	7 (35)	
D1	10 (50)	5 (25)	
D2	0	3 (15)	
D3	3 (15)	2 (10)	
E	5 (25)	0	
Revised Tokuhashi score, mean (SD)	7.2 (2.9)	5.8 (2.2)	0.1
SINS, mean (SD)	10.5 (1.9)	9.0 (2.0)	0.04
ESCCS, *n* (%)			0.1
0	2 (10)	0	
1a	2 (10)	0	
1b	0	1 (5)	
1c	1 (5)	1 (5)	
2	10 (50)	6 (30)	
3	5 (25)	12 (60)	0.6
Preoperative VAS, mean (SD)	5.7 (2.2)	6.0 (2.4)	0.6
Preoperative Barthel index, mean (SD)	65.5 (32.7)	41.0 (34.2)	0.06

Abbreviation: *SINS* Spinal Instability Neoplastic Score; ESCCS, epidural spinal cord compression scale; VAS, visual analogue scale

The results of statistical power for level of the primary lesion, modified Frankel classification, revised Tokuhashi score, SINS, ESCCS, preoperative VAS, and preoperative Barthel Index were 0.60, 0.86, 0.36, 0.10, 0.61, 0.05, and 0.44, respectively.

**Fig. 1:**
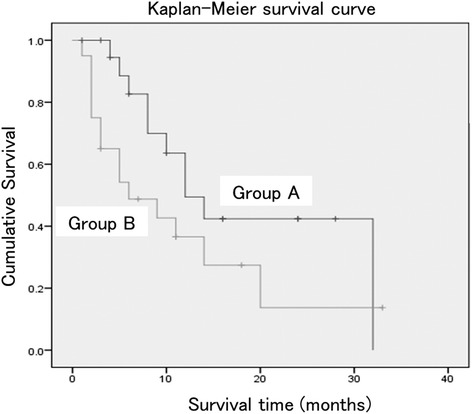
Kaplan-Meier survival curves for patients with metastatic spinal tumor treated with minimally invasive spine stabilization without (group A) and with (group B) decompression. The median survival times were 12.0 months (95% confidence interval, 12.6–24.8) in group A and 6.0 months (95% confidence interval, 6.0–16.6) in group B, with no significant difference between the groups (*P* = 0.09)

The median postoperative survival time determined using the Kaplan-Meier method was 12.0 months (95% confidence interval (CI), 12.6–24.8) in group A and 6.0 months (95% CI, 6.0–16.6) in group B, with no significant difference between the groups (*P* = 0.09) (Fig. 1). Patients in group A had a shorter mean operation time (*P* = 0.004) and lower mean intraoperative blood loss (*P* < 0.001) (Table 2). There were no significant differences in blood transfusion rates (*P* = 0.2), number of instrumented level (*P* = 0.06), or incidence of perioperative complications (*P* = 0.2) between the groups. The grade of postoperative paralysis (*P* = 0.1) and paralysis improvement (*P* = 0.09) did not differ significantly between groups A and B. The modified Frankel classification improved from D1 or D2 before surgery to D3 or E after surgery in 4 of 10 patients in group A (40%) and in 8 of 8 patients (100%) in group B (*P* = 0.01) (Tables 3 and 4). The postoperative Barthel Indexes were 85.8 and 60.3 (*P* = 0.06), and the postoperative VAS scores for pain were 1.8 and 1.4 in groups A and B (*P* = 0.6), respectively, with neither showing a significant difference between the groups. Significantly more patients in groups A were discharged to home (*P* = 0.02), and significantly fewer died in the hospital (*P* = 0.02). The rates of adjuvant chemotherapy (*P* = 0.1), radiotherapy (*P* = 1.0), BMA therapy (*P* = 0.2), and those of reaggravation of paralysis due to tumor enlargement at the treated level (*P* = 0.2) and reoperation (*P* = 0.1) did not differ significantly between groups A and B. In group A, paralysis reaggravated at the treated level in 3 of 14 (21.4%) and 2 of 6 (33.3%) patients who did and did not receive radiotherapy, with no significant difference between the subgroups (*P* = 0.5). In group B, these respective rates were 2 of 14 (14.3%) and 0 of 6 patients, again with no significant difference between the subgroups (*P* = 0.5).

**Table 2:** Summary of clinical results

Characteristic	Group A	Group B	*P* value
	(*n* = 20)	(*n* = 20)	
Operation time, mean (SD), minutes	166.5 (69.2)	232.1 (64.8)	0.004
Blood loss, mean (SD), mL	120.8 (155.3)	478.0 (493.9)	< 0.001
Transfusion, yes, *n* (%)	3 (15)	6 (30)	0.2
No. of instrumented level, mean (SD)	5.2 (1.5)	6.4 (2.3)	0.06
Perioperative complications, yes, *n* (%)	2 (10)	5 (25)	0.2
Epidural hemorrhage	0	2	
Massive bleeding (> 1500 ml)	0	1	
peritonitis	0	1	
Surgical site infection	0	1	
Instrumentation failure	1	0	
cerebral hemorrhage	1	0	
Postoperative modified Frankel category, *n* (%)			0.1
A	0	2 (10)	
B	0	1 (5)	
C	1 (5)	6 (30)	
D1	2 (10)	1 (5)	
D2	4 (20)	1 (5)	
D3	3 (15)	2 (50)	
E	10 (50)	7 (35)	
Neurological improvement by at least 1 grade or maintenance of grade E, *n* (%)	16 (80)	13 (65)	0.09
Postoperative Barthel index, mean (SD)	85.8 (24.3)	60.3 (39.7)	0.06
Postoperative VAS, mean (SD)	1.8 (2.6)	1.4 (1.4)	0.6
Postoperative course, *n* (%)			
Discharge to home	16 (80)	9 (45)	0.02
Transfer to hospice	4 (20)	6 (30)	0.4
In-hospital death	0	5 (25)	0.02
Additional adjuvant therapy, yes, *n* (%)			
Chemotherapy	14 (70)	8 (40)	0.1
Radiotherapy	14 (70)	14 (70)	1.0
Bone modifying agent	18 (90)	15 (75)	0.2
Local recurrence of paralysis	5 (25)	2 (10)	0.2
Revision surgery at local recurrence level	3 (15)	0	0.1

Abbreviation: *VAS* visual analogue scale

**Table 3:** Neurological recovery of group A on the modified Frankel scale

MFS	Number of cases before surgery	Number of cases after surgery						
		A	B	C	D1	D2	D3	E
A	0	0	0	0	0	0	0	0
B	0	0	0	0	0	0	0	0
C	2	0	0	1	0	0	0	1
D1	10	0	0	0	2	4	2	2
D2	0	0	0	0	0	0	0	0
D3	3	0	0	0	0	0	0	3
E	5	0	0	0	0	0	1	4
Total	20	0	0	1	2	4	3	10

**Table 4:** Neurological recovery of group B on the modified Frankel scale

MFS	Number of cases before surgery	Number of cases after surgery						
		A	B	C	D1	D2	D3	E
A	1	1	0	0	0	0	0	0
B	2	1	1	0	0	0	0	0
C	7	0	0	5	1	1	0	0
D1	5	0	0	0	0	0	1	4
D2	3	0	0	0	0	0	1	2
D3	2	0	0	1	0	0	0	1
E	0	0	0	0	0	0	0	0
Total	20	2	1	6	1	1	2	7

## Discussion

It is difficult to predict the prognosis of patients with metastatic spinal tumor preoperatively [2–6, 25], and the significance of palliative surgery for patients with a short life expectancy is controversial. The mean revised Tokuhashi scores in our patients were 5.8 and 7.2 in those treated with and without decompression, respectively. To perform palliative surgery in patients with such a short life expectancy requires consideration of the risk of surgery-induced complications, expected benefits, and medical costs [5, 24, 26]. However, there is an opinion that surgery should be performed [27], based on increased opportunities to receive postoperative adjuvant therapy by improving ADL, which may indirectly prolong survival. In our patients treated with and without decompression, we were able to perform adjuvant chemotherapy in 40 and 70%, respectively, radiotherapy in 70% in each group, and BMA therapy in 75 and 90%, respectively. MISt using PPS is advantageous in that adjuvant therapy can be performed early after surgery because the surgical wound is small and heals fast. Adjuvant therapies can be performed at a high rate, and this may have improved postoperative ADL in our patients.

Positioning of radiotherapy as adjuvant therapy is important. In a randomized controlled study comparing radiotherapy alone and combination therapy with palliative surgery and postoperative radiotherapy, the outcomes with the combination therapy were favorable [28]. Given that radiotherapy is important for local control of the tumor, we performed radiotherapy 2 weeks after surgery, as long as it is not inconvenient for treatment of the primary cancer. Radiotherapy was performed at a high rate (70%) in both groups, but there was no significant difference in outcomes with and without radiotherapy in both groups. This casts a slight doubt on the efficacy of postoperative radiotherapy, and further verification of this result is required.

In patients with metastatic spinal tumor, highly invasive palliative surgery may aggravate the general condition, and the opportunity for adjuvant therapy may be delayed or lost. Therefore, the invasiveness of palliative surgery needs to be minimized [9, 11–19]. Studies of outcomes of MISt alone [9, 13, 14, 17, 19] and comparisons of conventional posterior decompression and stabilization with MISt [11, 12, 15, 16, 18] have all concluded that MISt leaves a small surgical wound and has a short operation time and less intraoperative blood loss compared with the conventional method. However, differences in outcomes between MISt with and without decompression and the appropriateness of decompression have not been examined.

Massive bleeding can occur during preparation of the surgical field, application of decompression, and intratumor resection in palliative surgery. Resolving spinal instability without surgical stress due to intraoperative massive bleeding could facilitate early progression to adjuvant therapy. Posterior decompression and fusion surgery for a metastatic spinal tumor in palliative surgery has been recognized as the natural choice, but the advantages of not performing posterior decompression have not been discussed. In the current study, MISt without decompression included a significantly shorter operation time and reduced blood loss. These results showed that MISt without decompression is less invasive than MISt with decompression, and this may have led to higher rates of chemotherapy (70%), radiotherapy (70%), and BMA therapy (90%), although the differences were not significant, and the significantly higher rate of discharge to home and lower in-hospital mortality.

The disadvantage of MISt without decompression is that the spinal cord could only be indirectly decompressed. Those of reaggravation of paralysis due to tumor enlargement at the treated level and reoperation showed higher rate in group A, although the differences were not significant. Paralysis improved from a modified Frankel classification of D1 or D2 to D3 or E at rates of 100 and 40% in patients treated with and without decompression, with a significantly higher improvement rate after decompression. In the 6 of the 10 patients in group A whose modified Frankel classification did not improve to D3 or E after surgery, the mean Tokuhashi score and SINS were 5.1 and 11.6, respectively. Their ESCCS were 1a in one, 1c in one, 2 in two, and 3 in two. These results showed that the indication of MISt without decompression should be limited to mild paralysis of higher Tokuhashi score, lower SINS and lower ESCCS, and moderate nerve paralysis of D2 or severer requires decompression.

Regarding outcomes of palliative surgery with MISt for metastatic spinal tumor, many studies have focused on techniques, surgical stress, and short-term paralysis improvement, whereas only a few have examined postoperative survival, ADL, and quality of life (QOL) [12, 18]. Improvement of QOL at 30 days after surgery [12] and a median postoperative survival time of 11.3 months [18] have been reported. In the current study, the median postoperative survival times were 12.0 and 6.0 months, the mean postoperative VAS scores were 1.8 and 1.4, and the mean Barthel Indexes were 85.8 and 60.3 in group A and group B, respectively. None of these results differed significantly, indicating no significant effect of decompression on postoperative survival or improvement of ADL.

There are several limitations in this study. First, baseline characteristics such as main level of tumors, preoperative modified Frankel category, and SINS were different between the groups due to retrospective study. Because of the heterogeneity between the groups, there was a bias when the surgical procedure was selected since patients with severer spinal cord compression-induced paralysis were more likely to undergo decompression. Further, most patients received adjuvant chemotherapy and radiotherapy, respectively; the effect of surgical procedure versus effect of adjuvant therapy is difficult to dedifferentiate. Based on improvement of paralysis, the outcomes were similar in the two groups, but the use of MISt while spinal destruction by metastasis and paralysis are still mild may be better to maintain ADL and QOL. Thus, the first choice is MISt without decompression while paralysis is mild. Second, QOL was not fully evaluated. However, postoperative adjuvant therapy was introduced early in both groups and this may have led to a high rate of discharge to home and improvement of QOL. In addition, this rate was significantly higher in patients treated without decompression. In a large-scale study in Japan, cancer patients who chose to spend their last time at home lived longer than those who died in the hospital [29]. The postoperative survival time did not differ significantly between the groups in the current study, but it may have contributed to the improvement of QOL in patients treated without decompression. Third, the study was performed for a small number of patients at a single institution. The statistical power for baseline characteristics was under 0.8, excluding preoperative modified Frankel category. This result indicated that sample size was small for this study, and there could be not enough data available to show a difference between the groups. Therefore, verification of the results in a larger multicenter study is required.

## Conclusions

The postoperative survival time of patients with metastatic spinal tumor treated with MISt did not differ in treatment with or without posterior decompression. Patients treated without decompression had a shorter operation time, less blood loss, a higher rate of discharge to home, and lower in-hospital mortality, indicating a procedure with lower invasiveness. MISt without decompression was advantageous for patients with D3 or milder paralysis in the modified Frankel classification. The low invasiveness of MISt without decompression suggests that it is a more appropriate procedure for maintenance of ADL and QOL of patients. However, decompression is necessary in cases with D2 or severer paralysis.

## Abbreviations

ADL: Activities of daily living
BMA: Bone modifying agent
ESCCS: Epidural spinal cord compression scale
MISt: Minimally invasive spine stabilization
PPS: Percutaneous pedicle screws
QOL: Quality of life
SINS: Spinal Instability Neoplastic Score
VAS: Visual analogue scale
